# Decompressive Craniectomy in Traumatic Brain Injury: An Institutional Experience of 131 Cases in Two Years

**DOI:** 10.1089/neur.2020.0007

**Published:** 2020-10-07

**Authors:** Ana Cristina Veiga Silva, Matheus Araújo de Oliveira Farias, Luiz Severo Bem, Marcelo Moraes Valença, Hildo Rocha Cirne de Azevedo Filho

**Affiliations:** ^1^ Department of Neuropsychiatry and Behavioral Sciences, Federal University of Pernambuco, Recife, Brazil.; ^2^ School of Medicine, Federal University of Pernambuco, Recife, Brazil.; ^3^ Neurosurgery Department, Restauração Hospital, Recife, Brazil.

**Keywords:** decompressive craniectomy, intracranial hypertension, timing, trauma brain injury

## Abstract

Decompressive craniectomy (DC) effectively reduces intracranial pressure (ICP), but is not considered to be a first-line procedure. We retrospectively analyzed sociodemographic, clinical, and surgical characteristics associated with the prognosis of patients who underwent DC to treat traumatic intracranial hypertension (ICH) at the Restauração Hospital (HR) in Recife, Brazil between 2015 and 2016, and compared the clinical features with surgical timing and functional outcome at discharge. The data were collected from 131 medical records in the hospital database. A significant majority of the patients were young adults (age 18-39 years old; 75/131; 57.3%) and male (118/131; 90.1%). Road traffic accidents, particularly those involving motorcycles (57/131; 44.5%), were the main cause of the traumatic event. At initial evaluation, 63 patients (48.8%) were classified with severe traumatic brain injury (TBI). Pupil examination showed no abnormalities for 91 patients (71.1%), and acute subdural hematoma was the most frequently observed lesion (83/212; 40%). Glasgow Outcome Scale (GOS) score was used to categorize surgical results and 51 patients (38.9%) had an unfavorable outcome. Only the Glasgow Coma Scale (GCS) score on admission (score of 3-8) was more likely to be associated with unfavorable outcome (*p*-value = 0.009), indicating that this variable may be a determinant of mortality and prognostic of poor outcome. Patients who underwent an operation sooner after injury, despite having a worse condition on admission, presented with clinical results that were similar to those of patients who underwent surgery 12 h after hospital admission. These results emphasize the importance of early DC for management of severe TBI. This study shows that DC is a common procedure used to manage TBI patients at HR.

## Introduction

According to World Health Organization (WHO) estimates, each year around 5.8 million deaths occur worldwide that are due to or associated with traumatic injuries.^1^ Traumatic brain injury (TBI) stands out among other injuries for its significant contribution to mortality, disability, and health costs.^2,3^ Outside of suboptimal short- and medium-term TBI outcomes, these injuries have long-lasting effects that can both cause and accelerate other conditions such as neurological, psychiatric, and even non-neurological illness.^4^

The WHO recommended that the definition of trauma must be broadly constructed and based in terms of a chronic, if not definitive, disorder that is associated with permanent patient needs.^4^ The concept of TBI fits very well with this integrative definition, that the chronic nature of TBI is included, particularly given that more than 5 million people in the United States alone live with disabilities related to TBI.^5^

Severe head trauma can lead to brain swelling, increased intracranial pressure (ICP), reduced cerebral blood flow, inadequate oxygen delivery, ischemia, metabolic failure, and brain edema. Strategies to control ICP and maintain an adequate cerebral perfusion pressure (CPP) comprise a central principle in managing severe TBI.^6^ In some cases, hypertension is refractory to first- and second-level therapeutic measures,^7^ and requires emergency surgical intervention with decompressive craniectomy (DC). The DC procedure involves removal of portions of the cranial vault^8^ and subsequent durotomy to increase space that allows the swollen cerebral hemisphere to expand beyond normal cranial limits to immediately alleviate elevated ICP^9^ while avoiding internal herniation and brainstem compression.^2^ The increased space can lead to improved cerebral compliance, a reduction in ICP, and an increase in CPP that together increase both cerebral blood flow and cerebral microvascular perfusion.^10,11^

The role of primary DC in TBI remains controversial.^12^ Current guidelines discourage DC as a first-line therapy prior to exhausting clinical management.^13^ However, DC is sometimes used as a first-line treatment due to high demand, low resources, and lack of institutional facilities for delivery of adequate care.^14^

TBI is one of the main unresolved health problems worldwide, and is an endemic disease in low- and middle-income countries (LMICs).^15,16^ According to the WHO, in 2030 TBI will be the third-leading cause of death in the world.^1^ The long-term suffering and disability from the effects of TBI are a particularly important challenge for patients. TBI also presents a challenge for health care systems and incurs a substantial financial burden both for the patients' families and the general population.^1,17^

In Brazil, more than 1 million people are estimated to suffer a TBI annually. Of these, 20-30% of injuries can be classified as moderate or severe.^15,18^

The Restauração Hospital (HR) is a major trauma hospital and is the only public neurosurgical center that provides neurotrauma care in Recife, Brazil. The hospital has 830 bed capacity, 53 of which are in the general, pediatric, neurological, and burned intensive care unit (ICU); remaining beds are nursery and emergency beds. HR is an academic center and references tertiary transfer for all trauma emergencies, and includes neurosurgery, maxillofacial surgery, neurology, general surgery, a medical clinic, and orthopedics. HR sees about 35,000 annual hospitalizations of elective and emergency patients. The neurosurgery department performed nearly 3,200 neurological emergency procedures and about 400 DCs between 2015 and 2016.^18^

This study aimed to characterize the clinical and sociodemographic profile of patients with TBI submitted for DC at HR in order to identify prognostic factors associated with clinical outcome, timing of the procedure, and post-operative mortality and morbidity.

## Methods

A retrospective, descriptive, and observational study was performed using the HR hospital database. After obtaining hospital ethics committee approval (CAAE: 99813218.6.0000.5198) medical records for all patients with TBI who were submitted for DC at HR between January 2015 and December 2016 were assessed.

Patients <14 years-old were not included in the study. Medical records with incomplete information regarding patient identification, clinical data, or that lacked information about complementary tests, surgical description, or outcomes were excluded. Patients who had neurological deficits before the traumatic event and those having surgical lesions in other organs or systems were also excluded.

After admission to the hospital, patients with TBI are directed to a neurosurgeon, who conducts a primary assessment and stabilization regarding advanced trauma life support guidelines. At HR, DC for TBI is indicated primarily, with consideration of the physical examination, the patient's clinical signs and symptoms on admission, and radiological changes suggestive of increased ICP. Considering the high demand, there is no immediate access for most patients to ICU beds, nor is an ICP monitor readily available. The standardized technique for performing DC is a large, fronto-temporo-parietal hemicraniectomy (15 × 12 cm minimum) with middle fossa decompression and dural opening.

### Statistical analysis

The data analysis considered sociodemographic factors, mechanism of injury, Glasgow Coma Scale (GCS) score at hospital admission, pupillary alterations, lesions on computed tomography (CT) of the head, timing from hospital admission to surgery, use of ICP monitoring, duration of the surgery, post-surgical destination and length of stay, occurrence of cerebrospinal fluid (CSF) leakage, and surgical site infection. The neurological outcome was determined according to the Glasgow Outcome Scale (GOS) score at the time of discharge, and the duration of hospitalization was also analyzed. The level of significance was set at 5%. All statistical analyses were performed using SPSS software (version 18; SPSS, Inc.).

## Results

The present study reviewed 215 medical records of patients with TBI who underwent DC in HR between January 2015 and December 2016. Fifteen patients younger than age 14 years were excluded, and 69 were excluded for presenting, in association with TBI, surgical lesions in other systems, and/or incomplete registrations on medical records. A total of 131 patients were included in the analysis. The majority of patients in the study were young adults and male (average age 36 years-old, range 14-84 years-old). Patients were referred from all regions of Pernambuco state, and were transported distances of up to 300 km from other states. Road traffic accidents were the most frequent causative event of traumatic injury. Since significantly more traffic accidents involved motorcycles compared to other types of vehicles, motorcycle accidents were analyzed separately from other traffic accidents (Table 1).

**Table 1.** Sociodemographic Characteristics of 131 Patients with TBI Submitted for Decompressive Craniectomy

Variables	N	%
Sex	131	100
Male	118	90.1
Female	13	9.9
Age (years)	131	100
14–17	12	9.1
18–39	75	57.3
40–64	33	25.2
≥65	11	8.4
Distance from accident scene to hospital (km)	131	100
0–50	53	40.5
51–150	47	35.9
151–250	22	16.8
≥251	9	6.8
Mechanism of injury^a^	128	100
Road traffic accident	27	21.1
Motorcycle accident^b^	57	44.5
Fall	19	14.8
Assault	17	13.3
Firearm injury	8	6.3

^a^ The number of observations differs from the total sample as some information was not available (two patients were found unconscious, with physical signs but no record of traumatic event).

^b^ Motorcycle accidents were analyzed separately from other road traffic accidents as they were the main trauma mechanism identified.

TBI, traumatic brain injury.

The distribution of patient clinical status upon hospital admission and GCS score, pupil alterations, time from admission to surgical decompression, and brain CT scan lesions was collected from patient records (Table 2). Most of these patients had acute subdural hematoma (ASDH) and brain contusions, as single lesions found in 40% (83) and 32% (68), respectively, of all CT scans. Other findings were skull fracture (18/8.5%), subarachnoid hemorrhage (16/7.6%), epidural hematoma (12/5.7%), and ischemia and diffuse swelling (4/2% each).

**Table 2.** Distribution of Clinical Profiles for 131 Patients with TBI Submitted for DC

Variables	N	%
Trauma brain injury (Glasgow Coma Scale)^a,b^	129	100
Mild (13–15)	30	23.3
Moderate (9–12)	36	27.9
Severe (3–8)	63	48.8
Pupillary examination^a,c^	128	100
Both symmetric and reactive	91	71.1
Anisocoria–one reactive pupil	32	25
Mydriasis–no reactive pupils	5	3.9
Brain CT scan findings	131	100
Single lesions	67	51.1
Multiple lesions	64	48.9
Time from admission to surgery (h)^a^	127	100
<6	59	46.4
6–12	9	7.2
>12	59	46.4

^a^ The number of observations differs from the total sample as some information was not available.

^b^ Two patients had no GCS score because they were sedated at admission.

^c^ Pupillary examination was not done for three patients due to eye swelling and bruising.

CT, computed tomography; DC, decompressive craniectomy; GCS, Glasgow Coma Scale; TBI, traumatic brain injury.

Analysis of surgical results showed that most of the procedures were completed in about 2 h (65/50%) and had no watertight closure of the dura (72/57.1%) or installation of na ICP monitor (129/98.5%). The bone flap was discarded in 126 (96.2%) procedures. Two patients died intraoperatively, and 4 (3.1%) had immediate post-operative care in the ICU. Most patients stayed less than 5 days (57/131; 45.0%) in the post-operative care unit and had no CSF leakage (125/131; 95.4%) or surgical site infection (105/131; 80.2%). Of the 131 patients considered, 46 (35.1%) did not survive Table 3. The survivors were categorized as having favorable or unfavorable outcomes according to the GOS score.

**Table 3.** Distribution of Surgical and Post-Operative Results for 131 Patients with TBI Submitted for DC

Variables	N	%
Duration of the procedure (h)^a^	130	100
<2	35	26.9
2–4	92	70.8
≥4	3	2.3
Dural closure^a^	126	100
Yes	54	42.9
No	72	57.1
Post-operative destination^a,b^	129	SR
Post-anesthesia care unit	125	96.9
General intensive care unit	4	3.1
Length of stay on ICU/Post-operative care (days)	129	100
<5	57	44.5
5–15	29	22.3
>15	43	33.2
CSF leakage^a^	129	100
Yes	6	4.6
Surgical site infection^a^	129	100
Yes	26	20.2
Outcome–GOS score	131	100
Favorable (GOS 4–5)	34	26.0
Unfavorable (GOS 3–2)	51	38.9
Death (GOS 1)	46	35.1

^a^ The number of observations differs from the total sample because some information was not available.

^b^ Two patients died intraoperatively.

CSF, cerebrospinal fluid; DC, decompressive craniectomy; GOS, Glasgow Outcome Scale; ICU, intensive care unit; TBI, traumatic brain injury.

Table 4 summarizes the baseline demographic and clinical characteristics based on GOS at discharge. Only the GCS score from the admission evaluation was likely to be associated with patient outcome (*p*-value = 0.009). For the other variables, the independence test was not significant. However, this result may be biased because the categories were subdivided in order to better characterize the surgical results. In addition to GCS score, age (>64 years-old) and pupillary response to light (absent), were factors that were strongly associated with an unfavorable outcome, even death.

**Table 4.** Evaluation of Epidemiological and Clinical Characteristics According to Outcome (GOS at Discharge) for 131 patients with TBI Submitted for DC

Independent factors			Outcome: GOS					
	Death		Unfavorable		Favorable		Total	
	N	%	N	%	N	%	N	P-value
Sex							131	
Female	5	38.5	5	38.5	3	23.0	13	1.000^c^
Male	41	34.7	46	39.0	31	26.3	118	
Age (years)							131	
14–17	2	16.7	7	58.3	3	25.0	12	0.190^c^
18–39	24	32.0	29	38.7	22	29.3	75	
40–64	12	36.4	13	39.4	8	24.2	33	
≥65	8	72.7	2	18.2	1	9.1	11	
TBI mechanism^a^							128	
Motorcycle accident	16	28.1	25	43.8	16	28.1	57	0.185^c^
Assault	8	47.1	8	47.1	1	5.8	17	
Road traffic accident	12	44.4	7	25.9	8	29.7	27	
Firearm injury	3	37.5	1	12.5	4	50.0	8	
Fall	6	31.6	9	47.4	4	21.0	19	
Pupillary examination^a^							128	
Isocoria–both reactive	27	29.6	35	38.5	29	31.9	91	0.183^c^
Anisocoria–one reactive	14	43.8	13	40.6	5	15.6	32	
Mydriasis–neither reactive	3	60	2	40	0	—	5	
GCS on admission–TBI^a^							129	
Mild (13–15)	5	16.7	12	40.0	13	43.3	30	**0.009^b^**
Moderate (9–12)	10	27.8	16	44.4	10	27.8	36	
Severe (3–8)	31	49.2	22	34.9	10	15.9	63	
Timing of DC (h)^a^							127	
1–3	16	34	19	40.5	12	25.5	47	0.958^c^
>3–6	4	33.4	5	41.7	3	25.0	12	
> 6–12	3	33.3	5	55.6	1	11.1	9	
>12	22	37.3	21	35.6	16	27.1	59	

^a^ The number of observations differs from the total sample as some information was not available.

^b^ *P*-value generated from chi-square test for independence.

^c^ *P*-value generated from Fisher's exact test.

DC, decompressive craniectomy; GCS, Glasgow Coma Scale; GOS, Glasgow Outcome Scale; TBI, Trauma brain injury.

## Discussion

The results of the present study showed a direct relationship between available facilities and early DC because all procedures were conducted as primary treatments, and only two (1.5%) had ICP monitoring. These results are consistent with those of other studies of countries with low- and middle-income economies. Studies conducted at sites in which radiological changes were apparent in brain CT scans were shown to have a strong predictive value for intracranial hypertension (ICH).^19–21^

The sociodemographic outline for patients with TBI was traced from the results of the data analysis. Several previous studies obtained similar findings. Saade and colleagues,^21^ Khalili and associates,^6^ and Petgrave-Pérez and coworkers^22^ described the same social profile of patients with TBI and for some the same migratory trend from the country interior to urban centers was seen. Likewise, clinical profile and injury mechanisms of the patients in the present study was consistent with previous studies,^19,23,24^ thus confirming the need for more assertive traffic policies as a measure to prevent TBI.^25,26^

Vieira and colleagues^27^ also conducted a study in HR and found that DC without watertight duroplasty is not associated with a higher incidence of post-operative complications, frequency of CSF leakage, or infections. Moreover, DC patients had decreased surgical time by 31 min on average, resulting in a reduction in hospital costs for treatment of critically ill patients, especially those with severe TBI, since they configure greater demand. At HR there also is a partnership with the plastic surgery department for customized three-dimensional (3D) printed prostheses for cranioplasty.^28^

A variety of differently designed studies indicate that DC should significantly decrease the mortality of patients with severe TBI,^29–31^ but there still is no objective answer about which circumstances and which patients would realize the greatest benefit from DC. The main randomized controlled trials conducted to date, DECRA^33^ and Rescue ICP,^34^ did not clarify whether DC results in better clinical outcomes. TBI remains a substantial source of morbidity and mortality, mainly in areas with limited resources to adhere to Level 1 recommendation protocols, and particularly in those regions that have a higher burden of TBI mortality.^26,35^

In this context, the BEST trip trial^36,37^ and the consensus statement from the International Consensus Meeting on the Role of Decompressive Craniectomy in the Management of Traumatic Brain Injury^38^ stated oriented recommendations for the management of severe TBI in the absence of ICP monitoring (protocol based on clinical examination and imaging) and the role of DC in different scenarios. These guidelines are in place in advance of results for Rescue-ASDH (Randomized Evaluation of Surgery with Craniectomy for patients Undergoing Evacuation of Acute Subdural Hematoma), PRECIS,^39^ and also GNOS, a prospective, multi-center, ongoing international cohort study, which is focused on recruiting patients mainly from underdeveloped countries to generate a comprehensive picture of management and outcomes of patients who undergo emergency surgery to treat TBI.

## Conclusion

This study shows that the DC procedure is commonly used to manage patients with TBI at HR. The majority of these patients were young adult males involved in motorcycle accidents and were admitted in critical clinical conditions (GSC score 3-8) with at least one intracranial lesion on CT scan. GCS score on admission evaluation was found to be a strong predictor of patient outcome.

We also observed that critical patients (GCS score <9) who underwent surgery sooner after injury had the same clinical outcome as those who had a higher GCS score. The results may indicate that patients who underwent surgery sooner, despite having a worse condition on admission had similar clinical outcomes, thus emphasizing the importance of early DC in severe TBI management. However, clarification of whether these patients had, in most cases, a mass effect lesion requiring emergency intervention is needed.

No differences in the outcome results were observed with age, injury mechanism, pupil alterations, and timing of DC. The need to stimulate and improve registrations in medical records to avoid introduction of statistical inaccuracies in the patient database was also identified.

## Abbreviations Used

ASDH: acute subdural hematoma
CPP: cerebral perfusion pressure
CSF: cerebrospinal fluid
CT: computed tomography
DC: decompressive craniectomy
GCS: Glasgow Coma Scale
GOS: Glasgow Outcome Scale
HR: Restauração Hospital
ICH: intracranial hypertension
ICP: intracranial pressure
LMICs: low- and middle-income countries
TBI: traumatic brain injury
WHO: World Health Organization
